# Robert H. Ivy: The First Professor of Plastic Surgery and Cleft Palate Surgery Pioneer

**DOI:** 10.7759/cureus.71723

**Published:** 2024-10-17

**Authors:** Alexander H Chang, Adam Walchak, John D Potochny, Sameer A Patel

**Affiliations:** 1 Plastic and Reconstructive Surgery, Fox Chase Cancer Center, Philadelphia, USA; 2 Division of Plastic and Reconstructive Surgery, Penn State Health Milton S. Hershey Medical Center, Hershey, USA

**Keywords:** historical vignette, ivy society, pioneer, plastic surgery, robert h. ivy

## Abstract

Robert H. Ivy significantly influenced the field of plastic surgery through his innovative techniques, leadership, and dedication to the science and art of surgery. His dentistry background and military experience shaped his approach to maxillofacial and reconstructive surgery. Ivy's pioneering work in cleft lip and palate surgery, along with his use of local flaps, advanced the field of plastic surgery. Ivy was a key figure in the professionalization of plastic surgery; he played a crucial role in establishing the field as a distinct medical specialty, becoming the first-ever professor of plastic surgery. He was instrumental in founding major professional organizations and helped set the rigorous standards that govern plastic surgery today. His legacy lives on in the Robert H. Ivy Pennsylvania Plastic Surgery Society, a testament to his exceptional dedication to plastic surgery. Ivy's career exemplifies the power of innovation, leadership, and humility in advancing the practice of plastic surgery; his contributions continue to inspire and guide future generations of surgeons and surgical researchers.

## Introduction and background

**Figure 1:**
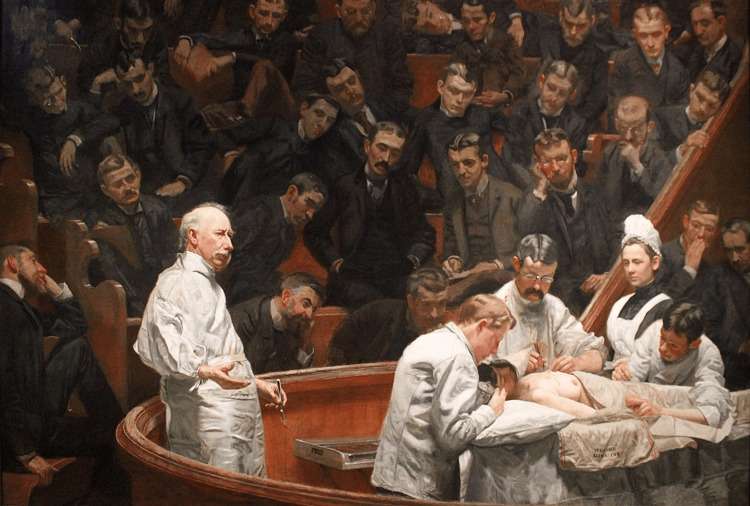
The Agnew Clinic by Thomas Eakins (1889) In a surgical amphitheater, Dr. Hayes Agnew performs a mastectomy, a procedure that would later be closely linked to the advancements in plastic surgery. At the time of the painting, Robert H. Ivy, who would later emerge as a pioneer in plastic and reconstructive surgery, was just eight years old [2].

Philadelphia has long been recognized as the birthplace of American medicine, home to many pioneering figures who have shaped the field of medicine and surgery. The city's rich medical history is rooted as far back as Benjamin Rush, a Founding Father, and Philip Syng Physick, often called the "Father of American Surgery," who emerged as a key figure in the early 19th century. These influential figures not only advanced medical science, but also laid the foundations for a vibrant medical community. Philadelphia's legacy of surgical innovation is further enriched by figures like Dr. Harvey Mutter, a very early pioneer of flap techniques [1]. This tradition is immortalized in Thomas Eakins's painting *The Agnew Clinic*, which captures the spirit of American medicine at the end of the 19th century (Figure 1) [2].

**Figure 2:**
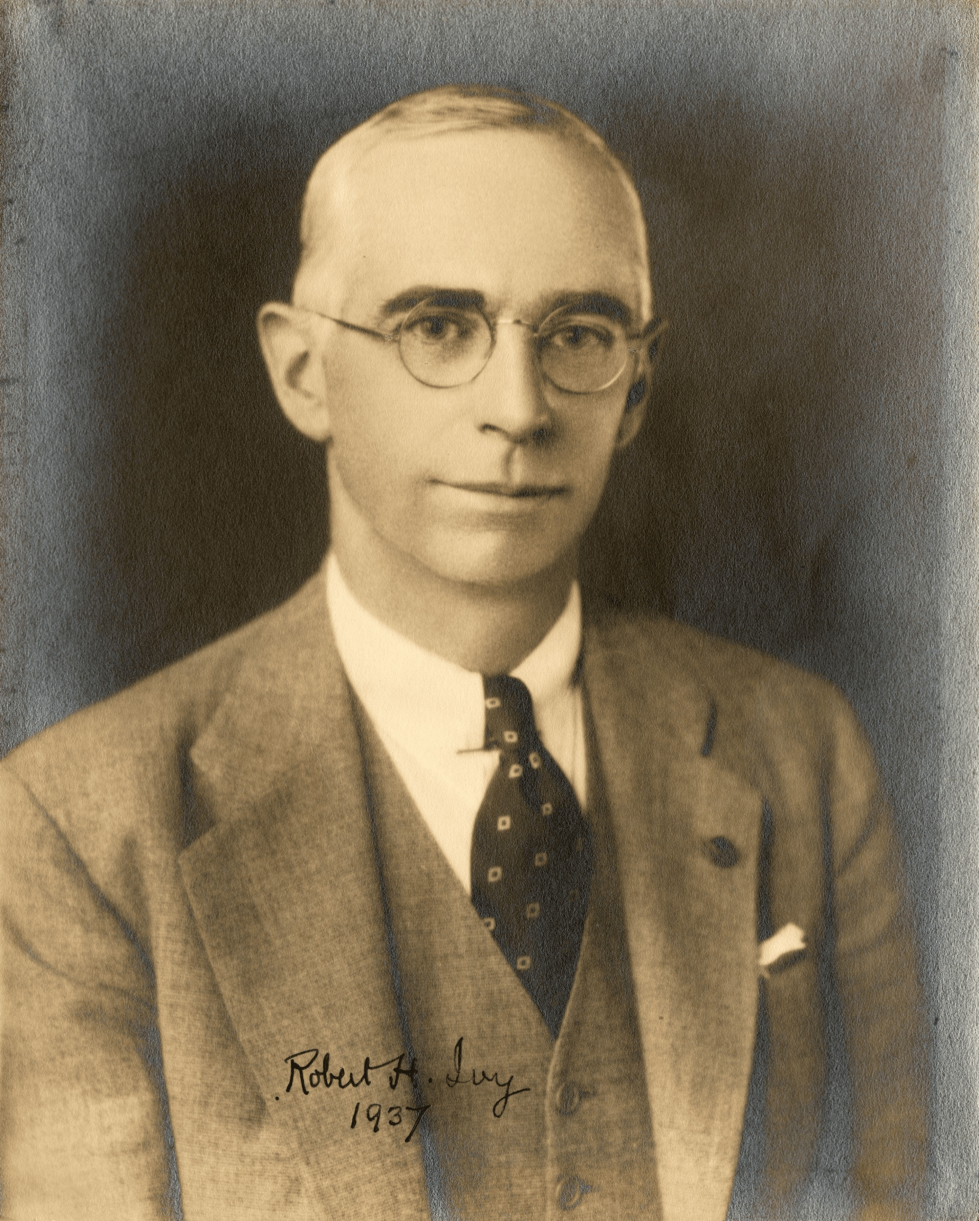
Robert H. Ivy 1937 Dr. Robert H. Ivy became the first physician in the United States to hold the title Professor of Plastic Surgery; his work laid the foundation for modern plastic and reconstructive surgery [3].

In this lineage of surgical giants, Robert H. Ivy emerges as a prominent figure in the early 20th century (Figure 2) [3]. His groundbreaking work in plastic surgery and his dedication to advancing the professionalization of the field reflect the innovative spirit that has long been a hallmark of Philadelphia's medical community. This vignette explores the life and contributions of Robert H. Ivy, examining his early influences, his impact on the development of plastic surgery, and his enduring legacy in surgical education and practice.

## Review

Early life and surgical career

Born in Southport, England, on May 21, 1881, Robert H. Ivy was educated in England before emigrating to the United States at the age of 17 years. He enrolled in the School of Dentistry at the University of Pennsylvania, graduating in 1902. Ivy then attended the School of Medicine from 1903 to 1907, during which time, Ivy went to China and took an excursion to Korea. Ivy chose to return to Philadelphia and pursue medicine, a decision he later reflected was a critical juncture in his life because of the geopolitical turmoil that would soon take over Asia. After completing his medical education, Ivy went on to complete a residency at the Episcopal Hospital of Philadelphia between 1907 and 1910 and subsequently opened an oral and plastic surgery practice in Philadelphia. Ivy also worked as an assistant to his uncle, Matthew H. Cryer, a Professor of Oral Surgery at the University of Pennsylvania’s School of Dentistry [4].

In 1917, Ivy joined the military as a captain in the Army Medical Corps, serving until 1919. During this time, he worked closely with Major Vilray Blair, who was responsible for organizing a section of plastic and oral surgery, a period Ivy later described as his apprenticeship [4]. Reflecting on his career decades later, Ivy attributed much of his success as a plastic surgeon to Blair, who saw to it that Ivy was stationed at desirable posts alongside other medical and surgical leaders [5]. Ivy fondly recounted his peer's encounter with Blair, who, when asked, "What is a plastic surgeon?" replied, "Just a damn fool who takes on things no one else will." The humorous quote encapsulated the daring spirit with which Blair and Ivy approached seemingly impossible surgical reconstructions; the two remained close friends for the rest of their lives [5].

While serving in the military, Ivy witnessed not only the devastating effects of the 1918 influenza epidemic but also the grim toll of World War I, particularly on the disfigured faces of soldiers returning from trench warfare. When he was assigned to Vichy, France, he collaborated with renowned surgeon Fernand Lemaitre and took charge of maxillofacial cases in specialized hospitals [4]. These experiences would lead Ivy to serve later on as a consultant at Walter Reed Hospital between World War I and World War II, where he would travel to Washington twice a month to consult on complex maxillofacial cases [6]. Such was his skill that the surgical teams, when faced with challenging cases, would "save it for Ivy” [4].

In 1920, Ivy met Harold Gillies, another pioneering figure in plastic surgery, during a presentation on the treatment of war injuries at the College of Physicians of Philadelphia. This encounter sparked a professional correspondence that spanned years; decades later Gillies even asked Ivy to name a device he had developed [7]. Such encounters with luminaries, combined with Ivy's military experience, reinforced his commitment to advancing the field of plastic surgery. Throughout his career, Ivy emphasized that plastic surgery involves the shifting or readjustment of tissues-a definition that underscores the foundational principles of the specialty he helped shape. Ivy responded to the surgical challenges of his time by integrating the works and studies of von Langenbeck and Parisian surgeon Victor Veau into his own practice, which he limited to plastic surgery cases, something only a few surgeons did at that time [4].

In his autobiography, Ivy reflects, "I have been asked the question, 'What do you consider your most important professional contributions?' It does not appear that I have done much of anything important. My name is not attached to any particular operation or method of treatment; I am not known for specially good results in any category of cases. I have published nothing on original scientific research" [4].

Ivy's words reflect overwhelming amounts of humility, characteristic of great surgeons who prioritize the advancement of their craft over personal recognition. While Ivy may downplay his original contribution, his impact on plastic surgery is undeniable as evidenced by the "Ivy Loop," a source of historical confusion to many. The term Ivy loop refers to dental wire loops used for intermaxillary fixation of maxillary fractures; although often attributed to Ivy, they were actually developed by Colonel Robert T. Oliver [4]. Ivy himself wished the eponymous loops referred to the special plastics technique called a tubed pedicle flap [8]. Regardless of this historical discrepancy, Ivy's name remained prominent and synonymous with innovation and influence in plastic surgery.

Ivy's most enduring legacy in plastic surgery is his pioneering work in the surgical correction of the cleft lip and palate. Such congenital deformities present significant challenges not only in terms of physical appearance but also in essential functions such as speech, swallowing, and breathing. At the turn of the 20th century, cleft lip and palate surgery was still in its infancy, and outcomes were often unpredictable, with high rates of complications and the need for multiple corrective procedures. Ivy was also a strong advocate for the use of grafts in reconstructive surgery, particularly in the repair of complex facial defects and traumatic injuries involving the oromaxillofacial region [8].

Ivy developed several flap techniques that allowed for the transfer of tissue from adjacent areas of the body to cover defects while preserving function and aesthetics. His contributions in this area were instrumental in advancing the field of reconstructive surgery and remain influential in contemporary plastic surgical practice. Ivy’s approach to clinical practice was grounded in a deep understanding of the complex anatomy of the face and oral cavity [9].

Despite downplaying personal achievements, Ivy highlights his four contributions to the field of plastic surgery: 1) advocating for the recognition of plastic surgery as a distinct specialty; 2) establishing an early residency program at the Graduate Hospital of the University of Pennsylvania; 3) organizing a comprehensive care program for children with cleft lip and palate; and 4) advancing the *Journal of Plastic and Reconstructive Surgery*. These efforts underscore Ivy’s significant influence on the development and institutionalization of plastic surgery as a recognized medical field [4].

Professionalization of plastic surgery

Ivy was a tireless advocate for the professionalization of plastic surgery as a distinct medical specialty. During his career, plastic surgery was still an emerging field, with practitioners coming from diverse backgrounds (typically dentistry and surgery) and varying levels of training and expertise. Ivy recognized the necessity for a standardized approach to training and certification to ensure that plastic surgeons possessed the necessary skills and knowledge to provide the best possible care. Ivy was instrumental in founding the American Association of Plastic Surgeons (AAPS) in 1921. His vision for the AAPS was to create a professional community where the leading minds in plastic surgery could come together. So stringent were the requirements of the AAPS that two members had to be exempt from the requisite dual degree policy (MD and DDS or DMD) [10]. The AAPS quickly became a cornerstone of the specialty, promoting high standards of practice, fostering research, and facilitating the sharing of knowledge and innovations [10].

The 1930s are often considered the formative years of plastic surgery as a recognized subspecialty. During this period, only a few masters of the craft existed, scattered across major cities with figures like Ivy's mentor Blair in St. Louis, John Stage Davis in Baltimore, William Ladd in Boston, and Sir Harold Gillies in London [11]. In 1937, Ivy played a pivotal role in the founding of the American Board of Plastic Surgery (ABPS), the first certifying body for plastic surgeons in the United States [12]. As one of the founding members, Ivy helped establish the standards for board certification, which included rigorous training requirements, a comprehensive examination process, and continued education. The creation of the ABPS was a landmark achievement in the history of plastic surgery, setting the stage for the field's growth and evolution in the decades that followed [11]. Ivy's efforts helped ensure that plastic surgery would become a specialty practiced with the most rigorous technical standards.

Ivy was appointed Professor of Plastic Surgery at the Graduate Hospital, becoming the first ever professor of plastic surgery in 1943, a formal academic recognition of plastic surgery as a distinct specialty. Ivy’s leadership extended beyond the American Board of Plastic Surgery to several other professional organizations. He was a founding member of the American Society of Plastic and Reconstructive Surgeons (ASPRS), where he worked to promote the field and advocate for the interests of plastic surgeons [6]. With Ivy's numerous contributions to the field of plastic surgery, the ASPRS grew in membership and influence, becoming a leading voice in the medical community, known today as the American Society of Plastic Surgery (ASPS) [4].

Editorial work and medical literature

In addition to his clinical and professional achievements, Ivy was a prolific author and editor, contributing significantly to the medical literature in plastic surgery. He served as the editor of *Plastic and Reconstructive Surgery*, the premier journal in the field even to this day [4]. Ivy played a critical role in shaping the direction of research and disseminating new surgical techniques and findings. Through his editorial leadership, Ivy helped transform the journal from one that struggled to gather enough content into a highly selective publication and the leading forum for the exchange of ideas and innovations in plastic surgery [4].

Surgery entails an ongoing dialogue that spans centuries and continents, connecting surgeons as they boldly addressed the medical and surgical challenges of their times, with each quandary serving as an impetus for innovation. History is marked by relationships like those between Hippocrates and Herophilus, Galen and Ibn Sina, John Hunter and Edward Jenner, and William Halsted and Harvey Cushing. The conversations about anatomy, physiology, medicine, and surgery transcend generations, with surgeons building on the insights of their predecessors and contemporaries to push the boundaries of knowledge and the limits of techniques forward. Central to this never-ending dialogue is the assiduous discussion of techniques, the publication of case reports and studies, research presentations at conferences, and collaboration with other experts across the globe. Ivy exemplified this approach, actively contributing to the surgical community with his lantern slide presentations, published works, and lectures on innovative techniques. By engaging in these activities, Ivy not only advanced his own practice but also played a pivotal role in driving the continuous evolution of plastic surgery, ensuring that the latest advancements were shared and applied across the field.

Legacy

One of the most important professional organizations Ivy joined was a regional society that would take on his name. At the time, it was common for plastic surgeons to join national and regional societies [3]. A regional society started forming around the Philadelphia and Delaware Valley area; initially it was to be called the "Philadelphia Society of Plastic and Reconstructive Surgery" until someone suggested The "Robert H. Ivy Society" [4]. Because of his deep influence on plastic surgery, Ivy's name had become synonymous with excellence and innovation in plastic surgery. His close friend Dr. Henry P. Royster became the first president of the society which stood as a testament to Ivy's pioneering spirit and visionary leadership [6]. The society was established to advance medical and surgical research, foster communication within the Philadelphia plastic surgery community, and uphold the highest standards of clinical practice. To this day, the Ivy Society carries forward Ivy's legacy with unwavering dedication, appointing presidents, vice-presidents, and historians for their proven excellence in plastic surgery and leadership [13].

**Figure 3:**
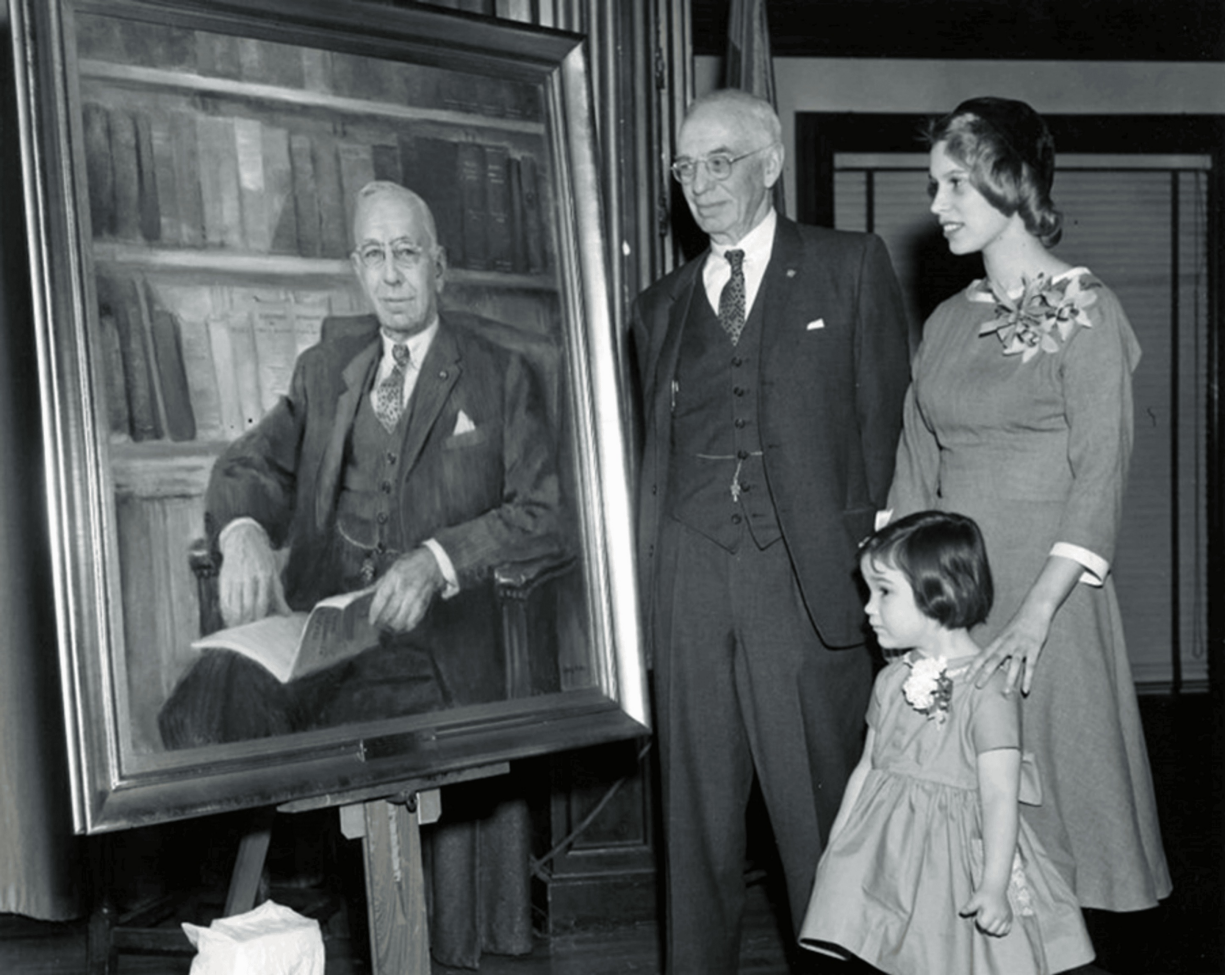
Unveiling of Dr. Robert H. Ivy's Portrait at Houston Hall, University of Pennsylvania, January 28, 1961 The portrait, painted by Philadelphia painter Agnes Allen, was presented by the Ivy Society to the University of Pennsylvania. Dr. Robert H. Ivy was 80 years old at the time. Standing next to him are his granddaughters [14].

Ivy's vision for plastic surgery is enshrined in the Society's four guiding principles: a relentless pursuit of innovation and research; a commitment to education and shared learning; a focus on the social, economic and psychological aspects of patient care; and an unwavering dedication to clinical excellence [6]. These principles ensure that Ivy's approach to plastic surgery endures, continuing to inspire new generations of plastic surgeons (Figure 3).

## Conclusions

Ivy’s career embodies the essence of Philadelphia’s innovative medical community. His contributions to plastic surgery, cleft lip and palate repair, skin grafting, and the establishment of plastic surgery as an academic discipline have had a profound and lasting impact. As a surgeon, educator, and leader, Ivy helped shape the future of plastic surgery, ensuring that Philadelphia remained at the cutting edge of surgical progress. His legacy ceaselessly inspires new generations of surgeons, establishing Ivy within the pantheon of American plastic surgery.

No vignette can truly capture the storied life of Robert H. Ivy, whose influence continues to foster a collaborative spirit among surgeons and surgical researchers, as evidenced by the Ivy Society. His notes, papers, and personal documents, preserved in collections at the Ivy Society, the College of Physicians, and the National Archives deserve to be reexamined to fully appreciate and understand the breadth of his contributions to plastic surgery. Every aspiring plastic surgeon can gain wisdom and draw inspiration from Dr. Robert H. Ivy's life, as his spirit continues to inspire excellence in surgical innovation in Philadelphia and throughout the surgical world.

## References

[1] Harris ES, Morgan RF (1994). Thomas Dent Mutter, MD: early reparative surgeon. Ann Plast Surg.

[2] (2024). Agnew Clinic. https://jstor.org/stable/community.14623616.

[3] Robert Henry Ivy (1881-1974), D.D.S. 1902, M.D. 1907, Sc.D Sc.D (2024). Robert Henry Ivy (1881-1974), D.D.S. 1902, M.D. 1907, Sc.D. (hon.) 1954, portrait photograph. Hon.) 1954, Portrait Photograph.

[4] Ivy RH (1962). A link with the past. https://archive.org/details/linkwithpast0000ivyr.

[5] Ivy RH (1971). Personal recollections of the organization and founders of the American Association of Plastic Surgeons. Plast Reconstr Surg.

[6] Blomain Blomain, E. (n.d History of Ivy Society (Part 1): Robert H. Ivy Society—The Man and the Physician. https://www.ivysociety.org/uploads/1/2/4/4/124459515/history_of_ivy_society__part_1_.pdf.

[7] Ivy RH (1969). The periwinkleshell operation. Plastic and Reconstructive Surgery.

[8] Tuerk M (1981). The Ivy loop. Ann Plast Surg.

[9] Ivy RH (1972). Iliac bone graft to bridge a mandibular defect. Forty-nine-year clinical and radiological follow-up. Plast Reconstr Surg.

[10] Ivy RH (1911). Applied anatomy and oral surgery for dental students.

[11] (2024). The History of the American Association of Plastic Surgeons: 1921-1996. https://aaps1921.org/multimedia/files/2018/History-1921-1996.pdf.

[12] Fox CG, Graham WP (1988). The American Board of Plastic Surgery, 1937-1987. Plastic & Reconstructive Surgery.

[13] Blomain Blomain, E. (n.d (2024). History of Ivy Society (Part 2): Robert H. Ivy Society. https://www.ivysociety.org/uploads/1/2/4/4/124459515/history_of_ivy_society__part_2_.pdf.

[14] Harding RL (1962). Robert H. IVY portrait presentation, University of Pennsylvania, Philadelphia, Pennsylvania, by The Robert H. Ivy Society and friends of Doctor Ivy. Plast Reconstr Surg Transplant Bull.

